# A Biomonitoring Study of Lead, Cadmium, and Mercury in the Blood of New York City Adults

**DOI:** 10.1289/ehp.10056

**Published:** 2007-07-23

**Authors:** Wendy McKelvey, R. Charon Gwynn, Nancy Jeffery, Daniel Kass, Lorna E. Thorpe, Renu K. Garg, Christopher D. Palmer, Patrick J. Parsons

**Affiliations:** 1 Division of Environmental Health, New York, New York, USA; 2 Division of Epidemiology, New York City Department of Health and Mental Hygiene, New York, New York, USA; 3 Trace Elements Laboratory, Wadsworth Center, New York State Department of Health, Albany, New York, USA; 4 Department of Environmental Health Sciences, School of Public Health, University at Albany, State University of New York, Albany, New York, USA

**Keywords:** biomonitoring, blood, cadmium, fish, lead, mercury, methylmercury, NYC HANES, seafood, survey

## Abstract

**Objectives:**

We assessed the extent of exposure to lead, cadmium, and mercury in the New York City (NYC) adult population.

**Methods:**

We measured blood metal concentrations in a representative sample of 1,811 NYC residents as part of the NYC Health and Nutrition Examination Survey, 2004.

**Results:**

The geometric mean blood mercury concentration was 2.73 μg/L [95% confidence interval (CI), 2.58–2.89]; blood lead concentration was 1.79 μg/dL (95% CI, 1.73–1.86); and blood cadmium concentration was 0.77 μg/L (95% CI, 0.75–0.80). Mercury levels were more than three times that of national levels. An estimated 24.8% (95% CI, 22.2–27.7%) of the NYC adult population had blood mercury concentration at or above the 5 μg/L New York State reportable level. Across racial/ethnic groups, the NYC Asian population, and the foreign-born Chinese in particular, had the highest concentrations of all three metals. Mercury levels were elevated 39% in the highest relative to the lowest income group (95% CI, 21–58%). Blood mercury concentrations in adults who reported consuming fish or shellfish 20 times or more in the last 30 days were 3.7 times the levels in those who reported no consumption (95% CI, 3.0–4.6); frequency of consumption explained some of the elevation in Asians and other subgroups.

**Conclusions:**

Higher than national blood mercury exposure in NYC adults indicates a need to educate New Yorkers about how to choose fish and seafood to maximize health benefits while minimizing potential risks from exposure to mercury. Local biomonitoring can provide valuable information about environmental exposures.

Lead, cadmium, and mercury are naturally occurring metals, but most human exposure occurs as a consequence of human activities. Mounting awareness and concern about environmental pollutants and their adverse health effects have led to an increase in measures to protect the public from avoidable exposures.

Blood lead concentrations in the United States have declined dramatically since the 1970s because of the phaseout of leaded gasoline, the ban of lead in paint and consumer products, and the discontinuation of lead use in plumbing and domestically manufactured soldered cans (Annest et al. 1983; Brody et al. 1994; Muntner et al. 2003; Pirkle et al. 1994). However, even at current lower levels, evidence suggests that pre- or postnatal exposure can potentially impair a child’s intellectual function (Baghurst et al. 1992; Canfield et al. 2003; Gomaa et al. 2002; Lanphear et al. 2005; Schnaas et al. 2006). Low-to-moderate levels of lead exposure in pregnancy may also increase the risk of spontaneous abortion (Borja-Aburto et al. 1999) and preterm birth (Andrews et al. 1994). In the general adult population, lead exposure has been associated with elevated blood pressure and hypertension (Martin et al. 2006; Nash et al. 2003), kidney disease (Kim et al. 1996), peripheral arterial disease (Muntner et al. 2003; Navas-Acien et al. 2004), and cardiovascular and all-cause mortality (Menke et al. 2006; Schober et al. 2006).

Cadmium occurs naturally in some soils in addition to being deposited through emissions from mining operations and fossil fuel combustion, application of phosphate fertilizer or sewage sludge, and disposal of cadmium-containing products [Agency for Toxic Substances and Disease Registry (ATSDR) 1999]. Tobacco and food crops can take up cadmium from the soil, and shellfish can accumulate cadmium from the aquatic environment, making cigarette smoke and diet the principal sources of nonoccupational exposure in the United States. Epidemiologic evidence has linked relatively low-level cadmium exposure to renal dysfunction (Buchet et al. 1990; Jarup and Alfven 2004) and decreased bone mineral density (Åkesson et al. 2006; Staessen et al. 1999).

Exposure to mercury in the United States occurs predominantly from consumption of predatory fish that have bioaccumulated methylmercury from the aquatic environment (Bjornberg et al. 2003; Sanzo et al. 2001; Svensson et al. 1992). Methylmercury can cross the blood–brain barrier and interfere with functioning of the central nervous system. Children’s developing nervous systems appear to be most vulnerable [National Research Council (NRC) 2000]. Deficits in language, attention, and memory among children exposed *in utero* have been reported in studies from the Faroe Islands, New Zealand, and the United States (Crump et al. 1998; Grandjean et al. 1999; Oken et al. 2005). Because methylmercury easily crosses the placenta (Ask et al. 2002; Vahter et al. 2000) and concentrates in fetal blood (Stern and Smith 2003), exposure in women of reproductive age is of particular concern. There is also evidence that methylmercury exposure in adulthood might interfere with vision, motor function, and memory (Lebel et al. 1998; Yokoo et al. 2003) as well as increase the risk of cardiovascular disease (Virtanen et al. 2007).

Ongoing surveillance of exposure to toxic substances is essential for identifying and targeting high-risk groups, evaluating interventions, tracking exposure over time, and monitoring exposures during emergency situations. New York State (NYS) law requires that all children be tested for lead at 1 and 2 years of age. NYS law also requires clinical laboratories to report all blood lead levels and elevated levels of mercury and cadmium in blood or urine to the State Heavy Metals Registry. However, testing among adults is voluntary and, therefore, likely to overrepresent higher-risk groups, for example, those in certain occupations or who request tests because of known or suspected exposures.

In 2004 New York City (NYC) conducted the first-ever local health and nutrition examination survey [NYC Health and Nutrition Examination Survey (HANES)] in a representative sample of NYC adults. The survey measured blood concentrations of lead, mercury, and cadmium using a design that mirrored the National Health and Nutrition Examination Survey (NHANES). In the present article we describe blood metal concentrations by demographic and behavioral characteristics. Results will be used to prioritize public health actions in NYC, where demographics and environment differ in many respects from the United States as a whole.

## Methods

### Sample selection

The NYC HANES was a population-based, cross-sectional survey representing the civilian, noninstitutionalized adult population (20 years of age and older) residing in the five boroughs (counties) of NYC and was conducted between June and December 2004. Participants were recruited into the study using a three-stage cluster sampling design. The stages of sample selection were *a*) selection of census blocks, or groups of blocks; *b*) random selection of households within selected segments; and *c*) random selection of study participants within households. No oversampling of demographic groups was done.

### Data collection

Selected subjects were invited to any of four clinic sites in the boroughs of Manhattan, Brooklyn, the Bronx, and Queens for interview and blood collection. Using a face-to-face, computer-assisted personal interview, study participants were asked their age, sex, race/ethnicity (White; Black/African American; Asian/Hawaiian/ Pacific Islander, henceforth referred to as “Asian,” as there are few Hawaiians and Pacific Islanders in NYC; Native American/ Alaskan Native or other; and whether they consider themselves to be Hispanic/Latino), education, income, smoking status, place of birth, length of time in the United States, occupation, and consumption of fish or shellfish in the past 30 days. Current job information was categorized according to the Standard Occupational Classification System 2000 (U.S. Bureau of Labor Statistics 2000). The survey instrument was translated into Spanish; interviews in other languages were conducted using a staff or family member proxy or a telephone translation service. Blood specimens were collected by venipuncture using supplies provided specifically for trace metal measurements.

The NYC HANES protocol was approved by the NYC Department of Health and Mental Hygiene (NYC DOHMH) and the NYS Department of Health (NYS DOH) Institutional Review Boards. Study participants provided written, informed consent, and those who provided interview and laboratory data were remunerated $100 for their time. More information on data collection and protocols, as well as a detailed description of the study design, has been published (Thorpe et al. 2006).

Of the 4,026 households selected, 3,388 (84%) completed an eligibility interview. Of the 3,047 selected, eligible survey participants, 1,811 (59%) completed the interview and provided a blood sample, yielding an overall response rate of 50%.

### Laboratory methods

Specimens were shipped to the Wadsworth Center’s Trace Elements Laboratory at the NYS DOH, and stored at –80°C until analyzed. The Wadsworth Center’s Laboratory is certified under the federal Clinical Laboratory Improvements Amendments of 1988 (CLIA-88 1992) and holds an NYS DOH clinical laboratory permit for blood lead and trace elements.

Total mercury, lead, and cadmium were determined in whole blood using a PerkinElmer Sciex (PerkinElmer, Shelton, CT) ELANDRC Plu inductively coupled plasma–mass spectrometer (ICP-MS). The ICP-MS method has been validated for biomonitoring measurements (Palmer et al. 2006), and performance is assessed periodically through participation in four external quality assessment schemes, as well as the NYS DOH’s proficiency testing program for trace elements in whole blood. The ICP-MS instrument was calibrated for each of the metals using matrix-matched calibration standards. All calibration standards were traceable to the National Institute of Standards and Technology (NIST, Gaithersburg, MD).

Internal quality control (IQC) materials covering the range of exposure expected in the U.S. population were analyzed at the beginning and end of each batch of blood specimens and throughout each analytical run. The IQC samples were prepared in-house from whole blood obtained from lead-dosed animals and supplemented with inorganic cadmium, inorganic mercury, and methylmercury chloride. NIST Standard Reference Material 966 (Toxic Metals in Bovine Blood) was periodically analyzed throughout the study to maintain independent validation. Full details regarding the characterization of the IQC pools, including metal concentrations, and QC performance statistics have been described elsewhere (Palmer et al. 2006).

Method detection limits for lead, cadmium, and mercury were 0.05 μg/L, 0.09 μg/L, and 0.17 μg/L, respectively. Typical repeatability, or between-run imprecision, was 1.4–1.7% for lead, 3.1–4.1% for cadmium, and 2.6–3.7% for mercury. A repeat analysis was performed on any specimens exceeding the upper threshold of 4 μg/L for cadmium, 10 μg/dL for lead, or 10 μg/L for mercury. In addition 2.5% of all blood specimens were randomly selected for re-analysis.

### Variable definition

Education was dichotomized by collapsing adjacent categories with similar geometric means. This resulted in collapsing categories whose geometric means differed by no more than 6%. For the lead analyses, participants were dichotomized into having up to a high school diploma and some college or higher. For the mercury and cadmium analyses, participants were dichotomized into having less than a bachelor’s degree and a bachelor’s degree or higher.

Smoking status was defined as current, former, or never smoker. Ever smoking was defined as having smoked at least 100 cigarettes in one’s lifetime. Those who reported smoking 20 cigarettes or more per day (*n* = 83) were considered heavy smokers.

In addition to the broad race/ethnicity classifications of non-Hispanic White, non-Hispanic Black, non-Hispanic Asian, and Hispanic, we further classified as foreign-born Chinese any participant who was Asian and either reported a place of birth in China, Hong Kong, or Taiwan, or else requested a Chinese language interview (*n* = 93). The Chinese represent the largest subpopulation in the NYC Asian community.

We dichotomized blood metal concentrations using selected cut points. Mercury was dichotomized at ≥5 μg/L (the NYS reportable level) and ≥15 μg/L (the NYS investigation level). Lead was dichotomized at ≥5 μg/dL and ≥10 μg/dL, consistent with reporting in previous publications (Muntner et al. 2005; Schober et al. 2006).

### Statistical analysis

We applied sample weights to adjust for differential selection probabilities and survey nonresponse. Weights were poststratified to reflect the age, sex, race/ethnicity, and borough of residence breakdown of the NYC population (U.S. Census Bureau 2000). Weights are applied to all estimates presented here. We used SUDAAN software, version 9 (Research Triangle Institute, Research Triangle Park, NC) to account for the complex sampling design. Relative standard errors (RSEs) were computed for estimated means and prevalence. Estimates with RSEs >30% were noted as statistically unstable (National Center for Health Statistics 2005)

We calculated crude population geometric means for blood metal concentrations by taking the antilog of the mean of the natural log-transformed values. Upon visual inspection, logging the values made a substantial improvement toward the approximation of a normal distribution. We used the method of Korn and Graubard (1998) to estimate the 95th and 97.5th percentiles and their 95% confidence intervals (CIs) for blood metal concentrations. We provide the 95th percentile to allow direct comparison to NHANES estimates; we provide the 97.5th percentile because it is a clinical reference value used to interpret individual test results (National Committee for Clinical Laboratory Standards 2000).

We used *t*-tests to compare geometric mean and prevalence estimates across categories of nominal predictors. To test for trends across continuous predictors, we categorized income, education, years in the United States (among the foreign-born), and fish consumption variables into four ordinal levels (scored 1–4); we used age in continuous form. To test for trends across geometric means, we used the *p*-value associated with the beta coefficient from a crude linear regression of the natural logarithm of the metal concentration on the predictor. To test for a trend in prevalence, we used *p*-values associated with the beta coefficient from a crude binary linear model that regressed having an elevated metal (0 or 1) on the predictor (Fieldstein 1966). The binary linear model is equivalent to assuming that the prevalence (or proportion) increases linearly.

We fit multiple linear regressions of the log-metal concentrations on the predictor variables. We excluded persons categorized as “Native American or Non-Hispanic Other” race/ethnicity because of small numbers (*n* = 27), and those with missing covariate data (*n* = 77), in these models. To assess the relation between blood lead and cadmium, we added blood cadmium concentration to the adjusted model of lead concentration, and vice versa. The exponentiated model coefficients represent the proportional change in the arithmetic mean associated with each level of the predictor, relative to a referent level, adjusting for the other predictors in the model. We considered a result to be statistically significant if the 95% CI did not include one (*p* < 0.05).

## Results

The geometric mean blood lead concentration in NYC adults was 1.79 μg/dL (95% CI, 1.73–1.86). Sample levels all exceeded the limit of detection, and ranged between 0.33 and 37.5 μg/dL. There were eight people with blood lead concentrations > 10 μg/dL (statistically unstable population prevalence = 0.5%), and two exceeded the NYS adult investigation level of 25 μg/dL. Most of these eight were male (7) and born outside the United States (7). An estimated 4.8% of the NYC adult population had lead levels ≥5 μg/dL (95% CI, 3.7%–6.1%), including 12 women of reproductive age (20–49 years of age) (statistically unstable population prevalence = 1.4%). The 97.5th percentile for blood lead concentration overall was 6.29 μg/dL.

We describe blood lead results in Table 1. Geometric mean blood lead concentrations increased with age and decreased with income, education, and length of residence in the United States for the foreign-born (*p*-values for trend tests < 0.04). Blood lead concentrations were highest in heavy smokers (2.49 μg/dL), the foreign-born Chinese (2.66 μg/dL), and those working in construction and maintenance (2.86 μg/dL). Upon removal of the latter group, the geometric mean blood lead level in smokers decreased slightly to 2.00 μg/dL (95th percentile = 5.51 μg/dL), suggesting some confounding of the smoking association by occupation. Prevalence of current smoking among construction and maintenance workers was 45% compared with a citywide estimate of 23%.

The patterns of lead concentrations across population subgroups were similar after we adjusted for predictors simultaneously in a log-linear regression—with several exceptions. The crude association between decreasing income and increasing geometric mean blood lead was no longer apparent (*p*-value for trend test = 0.54), and former smokers had only 8% higher blood lead concentrations than never smokers (compared with a crude elevation of 26%). Age remained the strongest predictor of blood lead. Upon adding blood cadmium to the adjusted model, a 1-μg/L increase predicted a 22% elevation (95% CI, 17–28%) in mean blood lead concentration.

The geometric mean blood cadmium concentration in NYC adults was 0.77 μg/L (95% CI, 0.75–0.80) as shown in Table 2. All sample levels exceeded the limit of detection and ranged from 0.25 to 9.67 μg/L. There were four blood cadmium levels > 5 μg/L, two of which were measured in foreign-born Chinese males who also had blood concentrations of mercury > 15 μg/L (*n* = 1) or lead > 10 μg/dL (*n* = 1). No samples attained the NYS reportable level for cadmium of 10 μg/L, although the highest measured level of 9.67 μg/L came close. The 97.5th percentile for blood cadmium concentration overall was 2.49 μg/L.

Blood cadmium levels were most strongly associated with smoking status. Heavy smokers had the highest geometric mean cadmium concentration (1.58 μg/L) of all subgroups examined. However, the geometric mean among foreign-born Chinese New Yorkers (1.34 μg/L) exceeded that of current smokers (1.22 μg/L), even though the estimated prevalence of smoking in this population subgroup (21%) was not higher than that of the general adult population (24%).

Results from a multiple linear regression were consistent with the patterns of crude geometric means observed across population subgroups. Current smoking and Asian race/ethnicity remained the strongest predictors of elevated blood cadmium. Blood lead was a relatively strong predictor of blood cadmium. After adjusting for other predictors, a 5-μg/dL increase in blood lead concentration predicted a 17% elevation in blood cadmium concentration (95% CI, 5%–31%).

The geometric mean blood mercury concentration among NYC adults was 2.73 μg/L (95% CI, 2.58–2.89) as shown in Table 3. All sample values exceeded the limit of detection and ranged between 0.21 and 35.78 μg/L. About one quarter (24.8%; 95% CI, 22.2–27.7%), or 1.4 million NYC adults, had blood mercury concentrations equaling or exceeding the NYS reportable level of 5 μg/L. There were 54 participants (population prevalence = 2.8%) who exceeded the NYS investigation level of 15 μg/L. Women 20–49 years of age had a geometric mean blood mercury level of 2.64 μg/L and a 23.8% prevalence of blood mercury ≥5 μg/L, similar to the total population. The 97.5th percentile for blood mercury concentration overall was 15.37 μg/L

Frequent consumption of fish or shellfish was associated with increasing mercury levels (*p*-values for trend test < 0.01 for geometric mean and prevalence ≥5 μg/L) (Table 3). The geometric mean blood level in those who reported consuming fish or shellfish 20 times or more in the last 30 days (5.65 μg/L) was more than 4 times the level of those who did not consume fish or shell-fish (1.31 μg/L). Over half (56.2%) of those who reported consuming fish 20 or more times in the last 30 days had mercury levels ≥5 μg/L, almost eight times the prevalence in those who did not consume fish or shellfish (7.3%).

People born outside the United States had higher mercury levels than those born in the United States; however we did not see a trend toward increasing mercury concentration with shorter time in the United States as we did with lead levels. In contrast, those who had lived in the United States for > 10 years had a higher crude geometric mean blood mercury level than newer arrivals (*p* < 0.01).

The geometric mean blood mercury level in Asians was higher than other racial/ethnic groups (4.11 μg/L). The 95th percentile was among the highest (19.19 μg/L). The geometric mean in foreign-born Chinese New Yorkers was even higher (7.26 μg/L), surpassing that of all other subgroups we examined. Almost half of adult Asian New Yorkers (46.2%) had blood mercury ≥5 g/L. Among the 93 foreign-born Chinese New Yorkers in the survey, 68 had blood mercury concentrations ≥5 μg/L (population prevalence = 71.7%), and 19 of these were ≥15 μg/L (population prevalence = 20.0%).

Fish consumption was the strongest predictor of increasing blood mercury concentration in a multiple linear regression of log-mercury concentration on the predictors in Table 3. The increased blood mercury levels in Asians relative to Hispanics (referent group) dropped from a proportional increase of 1.86–1.29 after adjustment, whereas the association with higher income was attenuated less (down to 1.39 from 1.49). The crude geometric mean blood mercury remained lower in current smokers compared with never smokers (*p* < 0.01) and those with less education (*p*-value for trend test < 0.01), but the associations were attenuated and no longer statistically significant in the adjusted model.

## Discussion

Findings presented here from the nation’s first local HANES, conducted in NYC in 2004, suggest that there is variability in exposure to toxic metals across population subgroups. Blood lead increased most with age; blood cadmium increased most with cigarette smoking; and blood mercury was most strongly related to fish or shellfish consumption. New Yorkers who self-identified as Asian had the highest blood concentrations of all three metals compared with other racial/ethnic groups. Foreign-born Chinese New Yorkers, in particular, had higher mercury levels than the most frequent fish consumers, higher lead levels than the oldest New Yorkers, and higher cadmium levels than current smokers. The wide range of exposure to metals in a geographically contiguous but diverse urban population highlights the importance of local-level examination surveys in guiding public health actions.

**Figure 1:**
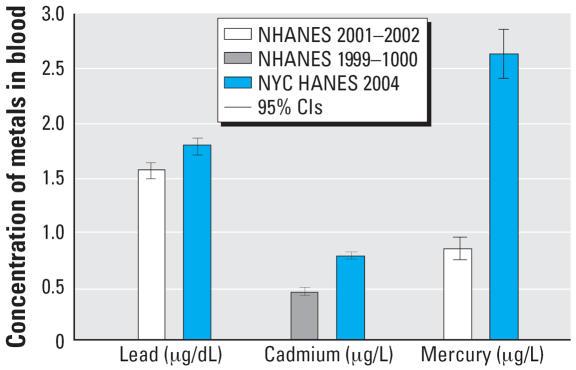
Geometric mean and 95% CI for blood lead, cadmium and mercury concentrations in adults residing in NYC compared with the United States overall, NYC HANES 2004, and NHANES 1999–2002 (CDC 2005a).*^a^* ***a***Blood mercury comparison for women age 16–49 years (NHANES) and 20–49 years (NYC HANES).

NHANES 1999–2002 (CDC 2005a) provided national estimates of blood mercury concentration for women 16–49 years of age. The geometric mean blood mercury concentration in our slightly older sample of NYC women 20–49 years of age (2.64 μg/L) is more than 3 times the NHANES 2001–2002 estimate (0.83 μg/L) [Centers for Disease Control (CDC) 2005a; Figure 1]. This elevation is consistent with a previous report of higher blood mercury levels in the Eastern coastal region of the United States relative to the United States as a whole (Mahaffey 2005).

Blood mercury levels were higher in NYC than nationally across similar levels of reported fish or shellfish consumption (Mahaffey et al. 2004). A possible explanation for this observation is that New Yorkers consume more heavily contaminated fish. A similar scenario may be occurring in the higher income groups, where mercury levels remain elevated even after adjustment for frequency of fish or shellfish consumption. Elevations in economically advantaged individuals may be due to consumption of more expensive fish, such as swordfish, which tend to be higher in mercury (Hightower and Moore 2003). However, even comparing people who reported no fish or shellfish consumption in the past 30 days, the geometric mean blood mercury concentration among New Yorkers was 3 times the national level (Mahaffey et al. 2004).

Blood metal concentrations among Asians have not routinely been reported from the NHANES because of sample size limitations. However, an analysis of 1999–2002 data identified the aggregate of Asians, Pacific Islanders, Native Americans and multiracial groups as having the highest mercury levels of all race/ethnicities (Hightower et al. 2006), similar to our findings. In NYC, fish consumption is the most likely explanation for the racial and ethnic differences in mercury exposure; consumption of at least 20 meals of fish or seafood in the last 30 days was highest in Asians (19%) compared with Whites or Blacks (5.5% each) and Hispanics (1.3%).

We are not aware of NHANES reports that describe elevated blood cadmium or lead in Asians, either alone or as an aggregate group, so we do not know whether the higher levels we measured among Asian New Yorkers mirror national data. Current smoking did not explain the higher cadmium or lead levels in Asians; in fact, prevalence of current smoking was slightly lower among Asian New Yorkers compared with the citywide estimate. Shellfish consumption is a possible source of the higher cadmium levels observed in Asians. Exposure could have occurred outside the United States as well, as cadmium and lead can remain in the body for decades, and body stores may serve as a source of subsequently measured metals in blood (Gulson et al. 1995; Nordberg and Kjellstrom 1979; Smith et al. 1996). In NYC, a large percentage (92%) of Asian adults are foreign-born (U.S. Census Bureau 2000).

The geometric mean blood lead concentration in NYC adults (1.79 μg/dL) is similar to the 2001–2002 national estimate (1.56 μg/dL; CDC 2005a; Figure 1). Despite declining trends (Muntner et al. 2005), current exposure levels have been associated with adverse health effects in children and adults (Canfield et al. 2003; Menke et al. 2006). In adults, nonoccupational lead exposure can occur during renovation of homes or other structures that used lead-based paints in the past. Residential remodeling was the likely source of exposure for the largest number of nonoccupational cases of blood lead ≥25 μg/dL reported to the NYS Heavy Metals Registry 2000–2005 (New York State Department of Health 2006). Other exposure sources included target shooting, ingestion (pica), lead-glazed pottery, soil, dust, and some imported food, spices and traditional medicines (ATSDR 2005; CDC 2005b; Saper et al. 2004). Cigarette smoke contains only small amounts of lead (ATSDR 2005), but our results are consistent with previous reports of positive associations between passive and active smoking and blood lead (Mannino et al. 2005; Shaper et al. 1982). It is possible that the association we observed was confounded by occupational lead exposure, as lead levels among current smokers decrease upon exclusion of persons who reported working in construction or maintenance.

The geometric mean blood cadmium concentration in NYC adults (0.77 μg/L) is slightly higher than the 1999–2000 national estimate for adults (0.47 μg/L; CDC 2005a; Figure 1). Though the difference appears to be statistically significant (judging from the nonoverlapping confidence intervals), the clinical or biological significance of a 0.3 μg/L elevation is not known. Decreased bone mineral density in older women has been associated with blood cadmium levels ≥1.1 μg/L (Alfven et al. 2000), which are typical of current smokers and the foreign-born Chinese in our survey. Cadmium is a constituent of cigarette smoke (ATSDR 1999), and the strong association between current smoking and blood cadmium provides further motivation to prevent smoking initiation and to promote smoking cessation.

Our findings have some limitations. Although the sample selection was designed to be representative of the NYC adult population, we cannot rule out the presence of bias, as the overall response rate was 50%. However, to correct for bias, sample weights incorporated information on age, sex, race/ethnicity, income, education, language spoken at home, and household size, obtained either directly from interview or from neighborhood census data. We also note that the NHANES interview and examination response rate for a similarly aged population in the NYC area in 2004 was only slightly higher, 58% (personal communication with the NHANES program), compared with the 55% response in the NYC HANES (response rates for blood collection component of the examination are slightly lower in both surveys).

Self-reported exposure data are limited by respondents’ memories and ability to answer questions. We do not know how accurately respondents were able to provide the number of times they ate fish or shellfish in the last 30 days. Furthermore, our questionnaire did not distinguish consumption of fish species according to mercury content. Consequently, confounding by contaminated fish and seafood consumption is likely to remain in our comparisons of mercury levels across population subgroups after adjustment for fish or shellfish consumption.

Laboratory methods for determining chemical exposures have become increasingly sensitive, so the detection of lead, mercury or cadmium in the blood of an adult does not necessarily imply a health risk. Findings are difficult to interpret in terms of public health impact, as reference doses are not necessarily meaningful threshold values for toxicity. The data we present attempt to describe exposures in the NYC adult population for the purpose of targeting intervention to high-risk groups and establishing baseline exposure levels.

A local HANES is an important source of information about the health of a community, particularly in the area of environmental exposures that are difficult—if not impossible—to assess without laboratory data, and that may vary across the nation. Our findings suggest that while NYC is keeping pace with national reductions in exposure to lead, exposure to mercury is elevated relative to national levels. The most significant source of exposure to mercury is likely to be fish consumption, implying a need to educate New Yorkers about how to choose fish to maximize health benefits while minimizing health risks. Asians may be at increased risk of exposure to mercury and other metals. Because lead and mercury are known to harm the developing nervous system and because both metals cross the placenta, it is critical that we support efforts to track and develop methods of intervention to reduce exposures in women of reproductive age. Our findings are also a reminder of the ramifications of failing to control mercury emissions into the environment.

## Figures and Tables

**Table 1:** Blood lead concentrations, geometric means, adjusted proportional change in means, 95th percentiles, and prevalence (≥5 μg/dL) in NYC adults by population subgroups.

Variable	No.^a^	Crude weighted geometric mean blood lead [μg/dL (95% CI)]	Adjusted proportional change in mean blood lead [μg/dL (95% CI)]^b^	Crude weighted 95th percentile blood lead [μg/dL (95% CI)]	No. with blood lead ≥5 μg/dL	Crude weighted % blood lead ≥5 μg/dL (95% CI)
Total	1,811	1.79 (1.73–1.86)	—	4.81 (4.37–5.51)	78	4.8 (3.7–6.1)
Sex						
Male	762	2.14 (2.03–2.25)	1.36 (1.28–1.44)	5.87 (5.01–6.60)	53	7.4 (5.4–10.1)
Female	1,049	1.54 (1.48–1.62)	1.00 (reference)	3.88 (3.65–4.36)	25	2.5 (1.6–3.9)
Age (years)						
20–39	903	1.42 (1.35–1.49)	1.00 (reference)	3.71 (3.23–4.24)	19	1.8 (1.1 –2.8)
40–59	673	1.99 (1.89–2.10)	1.38 (1.31–1.47)	5.56 (4.31–6.29)	38	5.7 (4.0–7.9)
≥60	235	2.40 (2.23–2.58)	1.70 (1.54–1.87)	5.77 (4.58–6.95)	21	9.1 (5.7–14.2)
Race/ethnicity^c^						
White, non-Hispanic	529	1.89 (1.77–2.01)	1.15 (1.05–1.26)	4.38 (4.23–5.26)	16	4.0 (2.3–6.8)
Black, non-Hispanic	390	1.73 (1.63–1.84)	1.08 (1.00–1.17)	5.56 (4.08–6.51)	22	6.5 (4.2–10.0)
Asian, non-Hispanic	231	2.14 (1.95–2.35)	1.31 (1.17–1.48)	5.51 (4.61–6.09)	16	7.3 (4.7–11.5)
Hispanic	630	1.62 (1.53–1.72)	1.00 (reference)	4.29 (3.78–5.02)	24	3.6 (2.2–5.8)
Place of birth						
U.S.	882	1.70 (1.62 –1.80)	1.00 (reference)	4.46 (4.20–5.56)	31	4.6 (3.1–6.8)
Outside U.S.	923	1.90 (1.81 –1.99)	1.14 (1.06–1.23)	4.97 (4.39–5.78)	47	5.0 (3.7–6.7)
Family income ($US)						
< 20,000	610	1.90 (1.79–2.01)	1.00 (reference)	5.32 (4.68–5.87)	39	6.5 (4.6–9.2)
20,000–49,999	566	1.76 (1.66–1.87)	0.96 (0.89–1.03)	5.01 (3.92–6.51)	22	5.4 (3.2–8.9)
50,000–74,999	256	1.70 (1.57–1.84)	0.96 (0.88–1.04)	4.24 (3.42–6.29)	15	3.0 (1.8, 5.0)
≥75,000	304	1.72 (1.60–1.85)	0.97 (0.89–1.06)	4.19 (3.69–4.65)		
Education						
High school diploma or less	862	1.95 (1.86–2.05)	1.09 (1.02–1.17)	5.76 (4.67–6.24)	52	6.8 (5.0–9.2)
Some college or more	941	1.68 (1.60–1.76)	1.00 (reference)	4.31 (3.89–4.73)	26	3.1 (2.0–4.8)
Smoking status						
Never smoked	1,036	1.61 (1.54–1.68)	1.00 (reference)	4.35 (3.79–5.30)	36	3.7 (2.5–5.5)
Former smoker	310	2.01 (1.88–2.16)	1.08 (0.99–1.17)	4.68 (4.03–6.72)	12	4.8 (2.5–9.0)^d^
Current smoker	449	2.09 (1.96–2.23)	1.31 (1.22–1.41)	6.00 (4.83–6.81)	30	7.3 (5.0–10.6)

a Totals do not all equal 1,811 because of missing data.

b The exponentiated βcoefficient from a log-linear multiple regression that includes all covariates in the table. Sample size for adjusted analysis is 1,707, after excluding study participants for whom covariate data are missing.

c Excludes 27 participants who self-classified as “other.”

d Statistically unstable population estimate.

**Table 2:** Blood cadmium concentrations, geometric means, adjusted proportional change in means, and 95th percentiles in NYC adults by population subgroups.

Variable	No.^a^	Crude weighted geometric mean blood cadmium [μg/L (95% CI)]	Adjusted proportional change in mean blood cadmium [μg/L (95% CI)]^b^	Crude weighted 95th percentile blood cadmium [μg/L (95% CI)]
Total	1,811	0.77 (0.75–0.80)	—	1.88 (1.73–2.07)
Sex				
Male	762	0.76 (0.73–0.79)	1.00 (reference)	1.95 (1.57–2.32)
Female	1,049	0.79 (0.76–0.82)	1.07 (1.03–1.11)	1.83 (1.73–2.01)
Age (years)				
20 to 39	903	0.72 (0.69–0.75)	1.00 (reference)	1.82 (1.58–2.06)
40 to 59	673	0.84 (0.80–0.89)	1.16 (1.11–1.22)	2.19 (1.90–2.52)
≥60	235	0.77 (0.73–0.81)	1.15 (1.08–1.23)	1.52 (1.32–1.63)
Race/ethnicity^c^				
White, non-Hispanic	529	0.73 (0.69–0.77)	1.04 (0.98–1.10)	1.71 (1.44–2.01)
Black, non-Hispanic	390	0.80 (0.75–0.86)	1.11 (1.04–1.18)	1.97 (1.74–2.48)
Asian, non-Hispanic	231	0.99 (0.90–1.09)	1.41 (1.27–1.57)	2.36 (1.65–3.43)
Hispanic	630	0.73 (0.71–0.76)	1.00 (reference)	1.73 (1.58–1.79)
Place of birth				
U.S.	882	0.76 (0.73–0.80)	1.00 (reference)	1.95 (1.75–2.32)
Outside U.S.	923	0.79 (0.75–0.82)	1.02 (0.98–1.07)	1.73 (1.52–2.19)
Family income ($US)				
< 20,000	610	0.86 (0.81–0.90)	1.00 (reference)	2.33 (1.90–2.75)
20,000–49,999	566	0.77 (0.73–0.80)	0.94 (0.89–0.99)	1.76 (1.49–2.22)
50,000–74,999	256	0.74 (0.69–0.79)	0.92 (0.86–0.99)	1.76 (1.51–2.71)
≥75,000	304	0.69 (0.65–0.74)	0.91 (0.85–0.97)	1.43 (1.17–1.71)
Education				
Less than bachelor’s	1,252	0.82 (0.79,0.85)	1.09 (1.04,1.15)	2.02 (1.87–2.4)
Bachelor’s or greater	551	0.69 (0.66,0.72)	1.00 (reference)	1.43 (1.28–1.57)
Smoking status				
Never smoked	1,036	0.66 (0.64–0.68)	1.00 (reference)	1.28 (1.20–1.34)
Former smoker	310	0.71 (0.67–0.74)	1.07 (1.02–1.12)	1.32 (1.10–1.58)
Current smoker	449	1.22 (1.15–1.29)	1.88 (1.78–1.99)	3.00 (2.65–3.49)

a Totals do not all equal 1,811 because of missing data.

b The exponentiated βcoefficient from a log-linear multiple regression that includes all covariates in the table. Sample size for adjusted analysis is 1,707, after excluding study participants for whom covariate data are missing.

c Excludes 27 participants who self-classified as “other.”

**Table 3:** Blood mercury concentrations, geometric means, adjusted proportional change in means, 95th percentiles, and prevalence (≥5 μg/L) in NYC adults by population subgroups.

Variable	No.^a^	Crude weighted geometric mean blood mercury [μg/dL (95% CI)]	Adjusted proportional change in mean blood mercury [μg/dL (95% CI)]^b^	Crude weighted 95th percentile blood mercury [μg/dL (95% CI)]	No. with blood mercury ≥5 μg/dL	Crude weighted % blood mercury ≥5 μg/dL (95% CI)
Total	1,811	2.73 (2.58–2.89)	—	11.03 (9.72–13.08)	431	24.8 (22.2–27.7)
Sex						
Male	762	2.67 (2.48–2.87)	0.95 (0.88–1.03)	10.70 (8.82–12.75)	195	25.5 (22.2–29.1)
Female	1,049	2.78 (2.61–2.97)	1.00 (reference)	11.31 (9.63–14.21)	236	24.3 (21.0–27.9)
Age (years)						
20–39	903	2.38 (2.20–2.56)	1.00 (reference)	9.54 (7.89–10.92)	179	21.5 (18.2–25.2)
40–59	673	3.23 (2.97–3.51)	1.30 (1.19–1.41)	15.31 (11.70–19.07)	198	30.3 (26.2–34.8)
≥60	235	2.71 (2.46–2.98)	1.22 (1.09–1.38)	8.07 (6.78–9.93)	54	22.3 (17.0–28.6)
Race/ethnicity^c^						
White, non-Hispanic	529	2.83 (2.62–3.07)	1.07 (0.96–1.20)	10.85 (9.36–14.21)	136	25.5 (21.5–29.9)
Black, non-Hispanic	390	2.61 (2.36–2.88)	1.05 (0.94–1.16)	9.26 (7.77–12.26)	81	23.3 (18.6–28.9)
Asian, non-Hispanic	231	4.11 (3.24–5.21)	1.29 (1.03–1.61)	19.19 (14.03–23.95)	112	46.2 (36.6–56.1)
Hispanic	630	2.27 (2.11–2.43)	1.00 (reference)	8.46 (7.03–9.93)	96	16.7 (13.5–20.5)
Place of birth						
U.S.	882	2.39 (2.24–2.56)	1.00 (reference)	8.32 (7.59–10.72)	152	18.9 (15.9–22.4)
Outside U.S.	923	3.15 (2.89–3.42)	1.38 (1.24–1.53)	13.39 (10.80–17.00)	279	31.3 (27.2–35.9)
Family income ($US)						
< 20,000	610	2.39 (2.17–2.63)	1.00 (reference)	9.84 (7.96–14.39)	113	19.3 (15.1–24.3)
20,000–49,999	566	2.55 (2.36–2.76)	1.05 (0.96–1.15)	9.89 (7.69–11.20)	116	20.5 (17.0–24.4)
50,000–74,999	256	3.02 (2.70–3.38)	1.21 (1.06–1.39)	11.19 (8.14–15.37)	75	30.4 (25.0–36.4)
≥75,000	304	3.56 (3.21–3.95)	1.39 (1.21–1.58)	14.69 (11.13–17.73)	111	37.2 (31.4–43.3)
Education						
Less than bachelor’s	1,252	2.54 (2.37–2.72)	1.00 (reference)	10.56 (8.50–13.39)	262	21.7 (18.6–25.1)
Bachelor’s or greater	551	3.16 (2.95–3.39)	1.07 (0.98–1.18)	11.54 (9.63–14.54)	169	31.5 (27.5–35.7)
Smoking status						
Never smoked	1,036	2.82 (2.65–3.01)	1.00 (reference)	10.72 (9.34–12.27)	257	26.6 (23.5–30.1)
Former smoker	310	2.83 (2.51–3.19)	0.96 (0.86–1.08)	11.76 (9.13–15.37)	86	25.6 (20.3–31.8)
Current smoker	449	2.43 (2.21–2.68)	0.93 (0.84–1.03)	11.34 (8.02–14.87)	84	19.8 (16.0–24.2)
Fish or shellfish consumption (last 30 days)						
Never	209	1.31 (1.14–1.50)	1.00 (reference)	5.39 (4.40–7.16)	14	7.3 (4.0–13.0)
Up to 9 times	1,216	2.60 (2.46–2.74)	1.90 (1.64–2.21)	9.34 (7.96–10.27)	237	20.5 (17.8–23.4)
10–19 times	255	4.25 (3.79–4.76)	2.87 (2.38–3.46)	19.19 (12.03–23.45)	111	44.1 (37.0–51.4)
20 times or more	114	5.65 (4.80–6.65)	3.70 (3.00–4.55)	18.31 (14.70–21.65)	65	56.2 (45.4–66.5)

a Totals do not all equal 1,811 because of missing data.

b The exponentiated βcoefficient from a log-linear multiple regression that includes all covariates in the table. Sample size for adjusted analysis is 1,707, after excluding study participants for whom covariate data are missing.

c Excludes 27 participants who self-classified as “other.”
